# Cost-effectiveness analysis of abiraterone, docetaxel or placebo plus androgen deprivation therapy for hormone-sensitive advanced prostate cancer

**DOI:** 10.31744/einstein_journal/2019GS4414

**Published:** 2019-02-25

**Authors:** Pedro Nazareth Aguiar, Pui San Tan, Sarah Simko, Carmelia Maria Noia Barreto, Bárbara de Souza Gutierres, Auro del Giglio, Gilberto de Lima Lopes

**Affiliations:** 1 Faculdade de Medicina do ABC, Santo André, SP, Brazil.; 2 Americas Centro de Oncologia Integrado, São Paulo, SP, Brazil.; 3 University of Oxford, Oxford, England, United Kingdom.; 4 School of Medicine, Universidade de Miami, Florida, FL, United States.; 5 MD Anderson Cancer Center, Houston, TX, United States.; 6 Universidade Paulista, São Paulo, SP, Brazil.; 7 Sylvester Comprehensive Cancer Center, Miami, FL, United States.

**Keywords:** Cost-benefit analysis, Drug therapy/economy, Hormone therapy/economy, Prostatic neoplasms/drug therapy, Drug costs, Placebos, Public Health, Análise custo-benefício, Tratamento farmacológico/economia, Hormonioterapia/economia, Neoplasias da próstata/tratamento farmacológico, Custos de medicamentos, Placebos, Saúde Pública

## Abstract

**Objective:**

To evaluate the cost-effectiveness of the addition of chemotherapy or abiraterone to androgen deprivation.

**Methods:**

We developed an analytical model to determine the cost-effectiveness of the addition of docetaxel or abiraterone *versus* androgen deprivation therapy alone. Direct and indirect costs were included in the model. The effects were expressed in Quality-Adjusted Life Years adjusted for side effects.

**Results:**

Compared to androgen deprivation therapy alone, the addition of chemotherapy and of abiraterone generated 0.492 and 0.999, respectively, in Quality-Adjusted Life Years. Abiraterone led to a Quality-Adjusted Life Years gain of 0.506 compared to docetaxel. The incremental costs per Quality-Adjusted Life Years were R$ 133.649,22 for docetaxel, R$ 330.828,70 for abiraterone and R$ 571.379,42 for abiraterone compared to docetaxel, respectively.

**Conclusion:**

The addition of chemotherapy to androgen deprivation therapy is more cost-effective than the addition of abiraterone to androgen deprivation therapy. However, discounts on abiraterone cost might improve cost-effectiveness.

## RESUMO

**Objetivo:**

Avaliar a relação custo-efetividade da adição de quimioterapia ou abiraterona à terapia de privação hormonal.

**Métodos:**

Um modelo analítico foi desenvolvido para determinar a relação custo-efetividade da adição de docetaxel ou abiraterona comparada à terapia de privação hormonal isolada. Custos diretos e indiretos foram incluídos no modelo. Os efeitos foram expressos em Anos de Vida Ajustados para Qualidade corrigidos pelos efeitos colaterais de cada terapia.

**Resultados:**

A adição de quimioterapia e de abiraterona à terapia de privação hormonal aumentou os Anos de Vida Ajustados para Qualidade em 0,492 e 0,999, respectivamente, em comparação à terapia de privação hormonal isolada. A abiraterona promoveu ganho de Anos de Vida Ajustados para Qualidade de 0,506 em relação ao docetaxel. O custo incremental por Anos de Vida Ajustados para Qualidade foi R$ 133.649,22 para o docetaxel, R$ 330.828,70 para a abiraterona e R$ 571.379,42 para a abiraterona comparada ao docetaxel.

**Conclusão:**

A adição de quimioterapia à terapia de privação hormonal é mais custo-efetiva que a adição de abiraterona à terapia de privação hormonal. Contudo, descontos no custo da abiraterona poderiam tornar esse tratamento mais custo-efetivo.

## INTRODUCTION

Prostate cancer is the most common neoplasm among men in Brazil, excluding non-melanoma skin cancers.^(^ ^1^ ^)^

Androgen deprivation therapy (ADT) used to be the standard treatment for newly diagnosed metastatic prostate cancer, also known as hormone-sensitive metastatic prostate cancer. In 2015, two important studies, STAMPEDE and CHAARTED, randomly enrolled patients to docetaxel plus ADT or ADT alone.^(^ ^2^ ^,^ ^3^ ^)^ CHAARTED enrolled 790 patients and found an overall survival (OS) benefit with the addition of docetaxel to ADT compared with ADT alone (median 57.6 months *versus* 44.0 months, respectively; hazard ratio – HR: 0.61; 95% confidence interval – 95%CI: 0.47-0.80). Similarly, STAMPEDE assigned 2,962 men and found an OS benefit with the addition of docetaxel to ADT compared with ADT alone (median 81 months *versus* 71 months; HR: 0.78; 95%CI: 0.66-0.93).^(^ ^2^ ^,^ ^3^ ^)^ Median OS seems to be higher in STAMPEDE compared with CHAARTED because men with high-risk localized prostate cancer were also eligible to STAMPEDE.^(^ ^2^ ^,^ ^3^ ^)^

In 2017, two other studies evaluated the combination of abiraterone plus ADT *versus* ADT alone for castration-sensitive metastatic prostate cancer.^(^ ^4^ ^,^ ^5^ ^)^ STAMPEDE-ABI randomized 1,917 patients and revealed that combinatory treatment improved OS by 37% when compared to ADT alone.^(^ ^4^ ^)^ Similarly, LATITUDE enrolled 1,199 men and showed that abiraterone plus ADT improved 3-year survival rate by 17%, as compared to ADT alone.^(^ ^5^ ^)^

Abiraterone is a steroidal CYP17A1 inhibitor that inhibits androgen synthesis in adrenal glands. This mechanism of action is interesting because adrenal gland is the second most important androgen-secreting gland (after testes) and is responsible for androgen secretion among men castrated by ADT. As a result, abiraterone has been studied for the treatment of castration-refractory metastatic prostate cancer before or after chemotherapy.^(^ ^6^ ^,^ ^7^ ^)^

CHAARTED, STAMPEDE and LATITUDE changed the mindset on prostate cancer treatment with their results, creating two additional standard therapies (docetaxel plus ADT, and abiraterone plus ADT) for hormone-sensitive metastatic prostate cancer. For the time being, due to the lack of data comparing abiraterone plus ADT versus docetaxel plus ADT, only indirect comparisons are possible.

The rising costs of antineoplastic therapies makes cost-effectiveness an important issue worldwide.^(^ ^8^ ^)^ With the prospective rise in the use of abiraterone and docetaxel plus ADT, it is important to understand their cost-effectiveness and how prostate cancer treatment costs might be affected.

## OBJECTIVE

To evaluate the cost-effectiveness of adding chemotherapy or abiraterone to androgen deprivation therapy *versus* androgen deprivation therapy alone, for patients with castration-sensitive metastatic prostate cancer.

The primary endpoint for this study was the incremental cost-effectiveness ratio defined as the incremental cost for each Quality-Adjusted Life Years gained with the new treatment.

## METHODS

**Figure 1:**
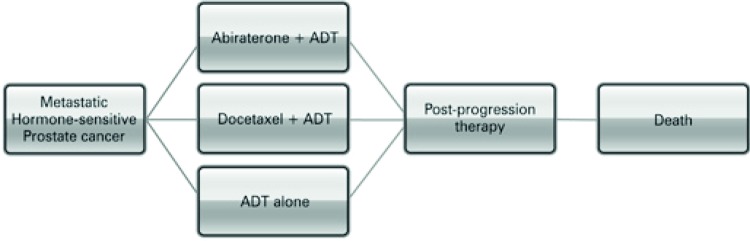
Analytic model of decision

We developed a descriptive-analytical model to evaluate the cost-effectiveness of the addition of abiraterone or docetaxel to ADT *versus* ADT alone, for patients with hormone-sensitive metastatic prostate cancer. The model considered three initial treatment options (ADT plus abiraterone, ADT plus docetaxel, and ADT alone) followed by post progression therapy and death ( Figure 1 ).

ADT: androgen deprivation therapy.

**Figure 2:**
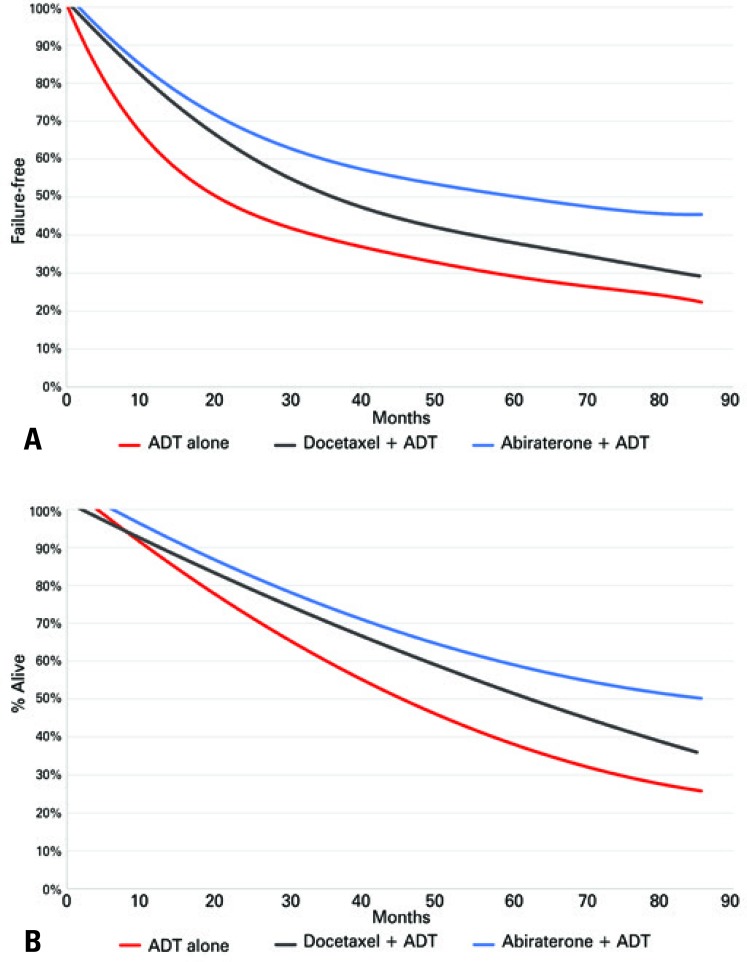
Survival estimates free of failure and overall survival. (A) Failure-free survival exponential estimative. (B) Overall survival exponential estimative

The efficacy of treatments was evaluated in Quality-Adjusted Life Years (QALY) using utility values for each health state (alive and without progression, alive after progression taking hormone therapy, alive after progression taking chemotherapy, and died). The utility values of each health state were extracted from literature.^(^ ^9^ ^)^ Failure-free survival (FFS) and OS of each arm in the model were extracted from the area under curve available in STAMPEDE clinical trials.^(^ ^3^ ^,^ ^4^ ^)^ The comparison between ADT plus abiraterone and ADT plus docetaxel used the outcomes retrieved from our recently published network meta-analysis.^(^ ^10^ ^)^ A lifetime horizon of 7 years was considered for FFS and OS using an exponential estimate ( Figure 2A and 2B ).

ADT: androgen deprivation therapy.

The adverse events caused by each treatment were considered in the calculation of QALY using disutility scores available in the literature.^(^ ^11^ ^,^ ^12^ ^)^

All drug acquisition costs were based on the Brazilian price indices accessed in December 2017.^(^ ^13^ ^)^ The costs of post-progression therapies were also considered. Costs related to monitoring costs, adverse event support and end-of-life care costs were considered based on the literature available.^(^ ^14^ ^,^ ^15^ ^)^

Based upon the World Health Organization recommendation, we considered a cost-effectiveness threshold of three times Brazilian Gross Domestic Product (GDP) *per capita* (approximately R$ 112.000,00 in 2018).^(^ ^16^ ^)^

We developed deterministic sensitivity analyzes to confirm robustness of our findings. Deterministic sensitivity analyzes considered FFS and OS 95% confidence intervals. In addition, we considered variations in abiraterone and docetaxel costs, indirect costs, and body surface area ( Table 1 ).

**Table 1:** Deterministic sensitivity analysis parameters

		95%CI	
Parameter	Mean deterministic	Lower value	Upper value
General			
Discount rate, %	10	NA	NA
	20	NA	NA
	30	NA	NA
	40	NA	NA
	50	NA	NA
Body surface area, m^2^	1.8	1.46	2.18
Occurrence of adverse events, %	Published	-10	+10
Costs			
Monitoring costs per month	R$ 448,00	R$ 358,00	R$ 537,00
End-of-life costs per case	R$ 1.034,00	R$ 827,00	R$ 1.240,00
Outcomes			
Progression-free survival utility	0,844	0,824	0,864
Post-progression survival utility @Hormone therapy	0,658	0,618	0,698
Post-progression survival utility chemotherapy	0,612	0,572	0,652
Survival			
HR on FFS docetaxel	0,62	0.54	0.70
HR on FFS abiraterone	0.29	0.25	0.34
HR on FFS abiraterone *versus* docetaxel	0.50	0.40	0.62
HR on OS docetaxel	0.73	0.59	0.89
HR on OS abiraterone	0.63	0.52	0.76
HR on OS abiraterone *versus* docetaxel	0.81	0.66	1.00

Currency rate: US$ 1.00 to R$ 3,75. 95%CI: 95% confidence interval; HR: hazard ratio; FFS: failure-free survival; OS: overall survival.

## RESULTS

### Cost-effectiveness

In the base-case, the addition of docetaxel to ADT compared to ADT alone led to a QALY gain of 0.492. The incremental cost was R$ 133.649,22 per QALY.

The addition of abiraterone to ADT increased the QALY by 0.999 compared to the ADT alone. The incremental cost per QALY was R$ 330.827,70.

Abiraterone plus ADT improved QALY by 0.506 compared to docetaxel plus ADT, with an incremental cost of R$ 571.379,42 per QALY. The base-case findings are summarized in table 2 .

**Table 2:** Summary of base-case analysis

Parameters	Abiraterone + ADT *versus* ADT	Docetaxel + ADT *versus* ADT	Abiraterone +ADT *versus* docetaxel + ADT
Number of cycles	34	5.614	NA
Drug cost	R$ 378.549,00	R$ 54.336,00	NA
Adverse events costs	R$ 2.042,00	R$ 3.526,00	NA
Post progression drugs costs	R$ 70.455,00	R$ 103.446,00	NA
End-of-life costs	R$ 112,00	R$ 172.00	NA
Monitoring costs	R$ 14.808,00	R$ 15.256,00	NA
Total costs	R$ 465.966,00	R$ 176.738,00	NA
Mean FFS, months	52.81	44.85	NA
Mean PPS, months	8.95	11.13	NA
Mean OS, months	61.76	55.98	NA
Utility	4.21	3.72	NA
AEs	-0.029	-0.052	NA
QALY gain	0.999	0.492	0.506
LYS	1.09	0.61	0.48
ICER	R$ 330.828,70	R$ 133.649,22	R$ 571.379,42
Incremental cost per LYS	R$ 303.109,81	R$ 107.901,84	R$ 602.557,60

Currency rate: US$ 1.00 to R$ 3,75. ADT: androgen deprivation therapy; FFS: failure-free survival; PPS: post-progression survival; OS: overall survival; AEs: adverse events; QALY: Quality-Adjusted Life Years; LYS: life years saved; ICER: incremental cost-effectiveness ratio; NA: not assessed.

### Deterministic sensitivity analysis

**Figure 3:**
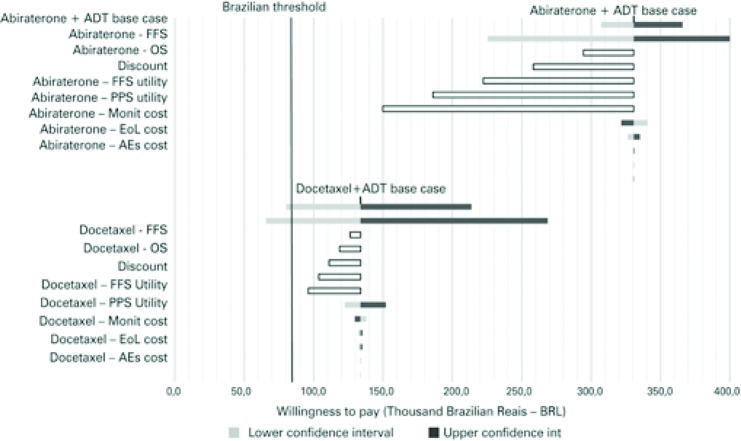
Tornado diagram for abiraterone plus androgen deprivation therapy or docetaxel plus androgen deprivation therapy *versus* androgen deprivation therapy alone

ADT: androgen deprivation therapy; FFS: failure-free survival; OS: overall survival; PPS: post-progression survival; EoL: End-of-life; AEs: adverse events.

The factors that had the greatest influence on cost-effectiveness were the confidence intervals for OS and FFS. In the case of abiraterone plus ADT *versus* ADT alone, price discounts when purchasing abiraterone was the factor that led to the greatest impact on the incremental cost and had a significant impact on cost-effectiveness ( Figure 3 ).

**Figure 4:**
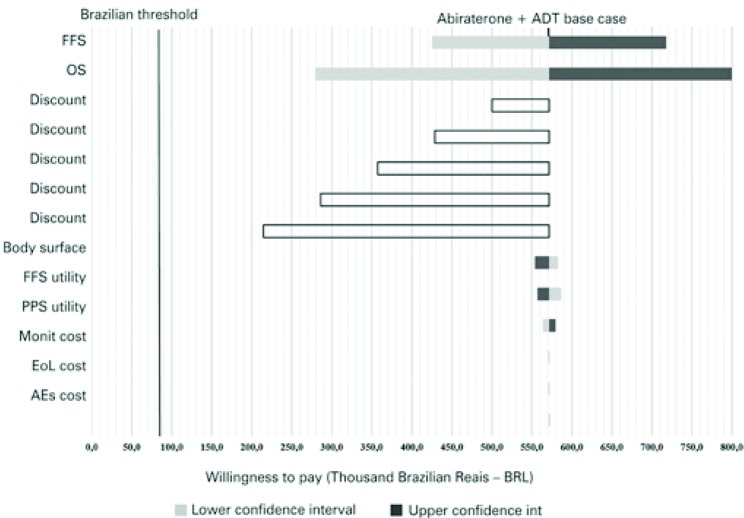
Tornado diagram for abiraterone plus androgen deprivation therapy *versus* docetaxel plus androgen deprivation therapy

ADT: androgen deprivation therapy; FFS: failure-free survival; OS: overall survival; PPS: post-progression survival; EoL: End-of-life; AEs: adverse events.

Considering data from the network meta-analysis to compare abiraterone plus ADT *versus* docetaxel plus ADT, the factors that had the greatest impact on cost-effectiveness were OS credibility intervals, and 50% discount on abiraterone acquisition cost ( Figure 4 ).

**Figure 5:**
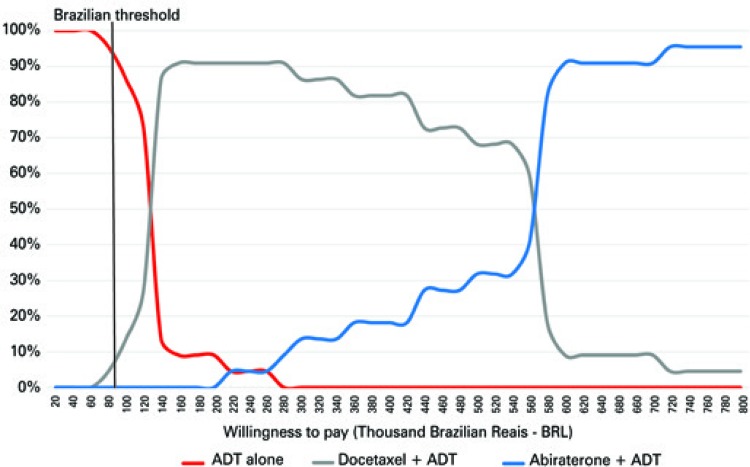
Probability of being cost-effective

Considering the World Health Organization (WHO) recommended threshold, ADT is the most cost-effective treatment in 94% of cases ( Figure 5 ). With an incremental investment of R$ 140.000,00, the combination of docetaxel plus ADT was the most cost-effective treatment in 91% of cases ( Figure 5 ). The acquisition cost of abiraterone made abiraterone plus ADT the most cost-effective treatment, only after an incremental investment of R$ 570.000,00.

ADT: androgen deprivation therapy.

## DISCUSSION

Although the findings of LATITUDE, STAMPEDE and CHARTED trials have expanded the standards of treatment for metastatic hormone-sensitive prostate cancer, their results may lead to a rise in the economic burden of this disease.

Despite cost-effectiveness being an important issue, there are relatively few studies in the literature focusing on this aspect of metastatic prostate cancer therapies.^(^ ^17^ ^)^ Currently, abiraterone is approved in Brazil only for men with castration-refractory metastatic prostate cancer. Interestingly, a systematic literature review found that most studies concluded that abiraterone is not a cost-effective solution for castration-refractory metastatic prostate cancer.^(^ ^17^ ^)^

In 2017, our group assessed the cost-effectiveness of docetaxel plus ADT compared to ADT alone and found that docetaxel should be cost-effective considering patients with newly-diagnosed high-volume metastatic prostate cancer.^(^ ^18^ ^)^

Now, we found that abiraterone plus ADT only became the most cost-effective therapy with an incremental investment of R$ 570.000,00.. Docetaxel plus ADT became the most cost effective in 91% of cases with an incremental investment of R$ 140.000,00. These findings show that − at current costs − docetaxel plus ADT is more cost-effective than abiraterone plus ADT. This conclusion may have a major impact on decision-making processes of the Brazilian healthcare system.

Another possibility could be the combination of both strategies: ADT plus docetaxel (six cycles) followed by ADT plus abiraterone until disease progression. This strategy should improve OS at most by combining benefits from docetaxel and abiraterone. In addition, the first six cycles of docetaxel plus ADT can decrease treatment cost compared with abiraterone plus ADT since diagnosis. However, this strategy has not been assessed in a randomized clinical trial yet and, consequently, cannot be considered in a cost-effectiveness analysis.

Pharmaceutical spending in Brazil has risen drastically over the past decade, with drug expenditures nearly tripling between 2006 and 2013 and increasingly growing.^(^ ^19^ ^)^

Currently, abiraterone is not available in the Brazilian public health system, which serves up to 75% of population.^(^ ^20^ ^)^ In order to improve patient’s access to abiraterone, discounts and price changes must be negotiated. Our study found that a 50% discount on abiraterone acquisition cost should decrease its incremental cost to became cost-effective, from R$ 570.000,00 to R$ 150.000,00.

One study assessed the cost-effectiveness of abiraterone plus prednisolone *versus* cabazitaxel plus prednisolone in patients with castration-refractory metastatic prostate cancer previously treated with docetaxel. This study found that, in the Brazilian private health system, abiraterone was both more effective at increasing QALYs and providing lower costs as compared to cabazitaxel.^(^ ^21^ ^)^ The study by Pereira et al.,^(^ ^21^ ^)^ is different from ours. First, they assessed the treatment for castration-refractory patients previously treated with docetaxel, while we assessed the treatment for newly diagnosed castration-sensitive disease (median duration of abiraterone therapy 6 months *versus* 34 months, respectively). Second, they compared abiraterone to cabazitaxel, a third-generation taxane that is as expensive as abiraterone is (R$ 10.071,00 per cycle and R$ 10.625,00 per cycle, respectively).

To enhance allocation of scarce resources, further studies are necessary to identify biomarkers for castration-sensitive patients, who will benefit most from abiraterone plus ADT.

## CONCLUSION

We concluded that the addition of chemotherapy to androgen deprivation therapy is more cost-effective than the addition of abiraterone to androgen deprivation therapy. Discounts on abiraterone cost may make this treatment more cost-effective.

New studies may help identify biomarkers for patients who will benefit most from each treatment (androgen deprivation therapy alone, androgen deprivation therapy plus docetaxel, and androgen deprivation therapy plus abiraterone) improving allocation of resources.
